# Effect of a Pharmacy-based Centralized Intravenous Admixture Service on the Prevalence of Medication Errors: A Before-and-After Study

**DOI:** 10.1097/PTS.0000000000001047

**Published:** 2022-06-30

**Authors:** Janique G. Jessurun, Nicole G.M. Hunfeld, Joost van Rosmalen, Monique van Dijk, Patricia M.L.A. van den Bemt

**Affiliations:** From the Departments of ∗ Hospital Pharmacy; † Intensive Care; ‡ Biostatistics; § Epidemiology, Erasmus MC, University Medical Center Rotterdam, Rotterdam; ∥ Department of Clinical Pharmacy and Pharmacology, University Medical Center Groningen, Groningen; ¶ Internal Medicine, Section of Nursing Science, Erasmus MC, University Medical Center Rotterdam, Rotterdam, the Netherlands.

**Keywords:** medication errors, patient safety, medication safety, intravenous admixture, intravenous medication, intravenous admixture preparation error

## Abstract

Intravenous admixture preparation errors (IAPEs) may lead to patient harm. The primary aim of this study was to assess the effect of a pharmacy-based centralized intravenous admixture service (CIVAS) on IAPEs.

**Methods:**

We conducted a before-and-after study in 3 clinical wards before CIVAS implementation and in the CIVAS unit 18 months after implementation. Intravenous admixture preparation error data were collected by disguised observation. The primary outcome was the proportion of admixtures with 1 or more IAPEs. Secondary outcomes were the type and potential severity of IAPEs, noncompliance to hygiene procedures, and nursing staff satisfaction with the CIVAS. The primary outcome was analyzed using a multivariable mixed-effects logistic regression model.

**Results:**

One or more IAPEs were identified in 14 of 543 admixtures (2.6%) in the CIVAS unit and in 148 of 282 admixtures (52.5%) in the clinical wards (odds ratio, 0.02; 95% confidence interval, 0.004–0.05). The most common IAPE types were wrong solvent or diluent (n = 95) and wrong volume of infusion fluid (n = 45). No potentially harmful IAPEs occurred in the CIVAS unit as opposed to 22 (7.8%) in the clinical wards. Disinfection procedures were better adhered to in the CIVAS unit. Overall nurse satisfaction with the CIVAS increased from a median of 70 (n = 166) 5 months after intervention to 77 (n = 115) 18 months after intervention (*P* < 0.001) on a 100-point scale.

**Conclusions:**

Centralized intravenous admixture service performed notably better than the clinical wards with regard to IAPEs and noncompliance to hygiene procedures. Nurses were satisfied with the CIVAS. Hence, the implementation of CIVAS is an important strategy to improve medication safety in hospitals.

Unsafe medication practices are a leading cause of preventable patient harm in healthcare settings.^1,2^ The preparation of intravenous admixtures especially carries a high risk of medication errors^3–5^ because it involves many steps.^3^ Moreover, erroneous preparation of intravenous admixtures poses an increased risk of causing patient harm due to the complete and immediate bioavailability after intravenous administration. Systematic reviews report high rates of intravenous admixture preparation errors (IAPEs), mainly occurring in clinical wards.^5–8^ For example, rates of wrong-drug errors varied substantially between studies from 0% to 5% and of wrong dose from 0% to 33%, whereas rates of harmful errors varied between 0% and 64%.^6^ Nonetheless, preparation of intravenous admixtures in clinical wards is still common.^9^ Centralizing intravenous admixture preparation in hospital pharmacies has been explored as a potential strategy to improve patient safety.^9–12^ The few centralization studies that have examined patient safety–related outcomes, such as IAPEs,^13–16^ showed promising results with respect to reduction in rates of concentration errors (from 55% to 38% and 22% to 5%),^13,14^ calculation errors (from 1% to 0%),^13^ and microbiological contamination (from 1% to 0%, 22% to 1%, and 2% to 0%).^15,16^ However, these studies have relevant limitations with regard to generalizability, for example, small sample sizes^14,16^ and inclusion of simulated preparations solely intended for research purposes,^14,16^ specific wards such as intensive care units,^13,14,16^ and few IAPE types.^13–16^ Furthermore, results of individual IAPE studies are hardly comparable because of clinical and methodological heterogeneity.^5,6,8^ Nursing staff satisfaction with the centralization of intravenous admixture preparation has not been studied yet. However, the effectiveness of newly implemented systems in preventing medication errors is likely to be related to the compliance and satisfaction of nursing staff with these systems.^17–20^

Therefore, we conducted a before-and-after study to assess the effect of the implementation of a pharmacy-based centralized intravenous admixture service (CIVAS) on the prevalence of IAPEs in a hospital setting. Secondary aims were to assess the effect on the type and potential severity of IAPEs; to assess the rates of noncompliance to hygiene, double-checking, and labeling procedures; and to determine nursing staff satisfaction with the CIVAS.

## METHODS

### Study Design

A prospective before-and-after study was conducted in Erasmus MC, University Medical Center Rotterdam in the Netherlands. The Medical Ethics Review Committee of Erasmus MC waived approval for this study (reference number MEC-2018-1170) in accordance with the Dutch Medical Research Involving Human Subjects Act. Nursing staff and pharmacy staff gave verbal consent for participation in this study. Data were treated confidentially according to the Dutch General Data Protection Regulation.

### Study Setting

Data collection took place from January 2018 to October 2019 in 3 clinical wards (hematology, internal oncology, and neurosurgery) and the hospital pharmacy of our institution. Three additional wards that had received the intervention before the initialization of this study, that is, hepatopancreatobiliary surgery, pulmonary medicine, and neurology, were included for the nursing staff satisfaction measurements.

### Usual Care

Before the implementation of the CIVAS, intravenous admixtures, except admixtures of chemotherapeutic medication and parenteral nutrition, were prepared by nursing staff in clinical wards. Details of setting characteristics are given in Table 1.

**TABLE 1:** Setting Characteristics

Characteristics	Clinical Wards Usual Care	Hospital Pharmacy CIVAS Period 1	Hospital Pharmacy CIVAS Period 2
Preparation environment	Workbench in medication room	LAF cabinet in clean room	LAF cabinet in clean room
Preparation, staff type	Nurse	Pharmacy technician	Pharmacy technician
Medication prescription software*	HiX Practocol	HiX	HiX
Medication preparation software*^†^	Electronic handbook	TgMed	HiX
Preparation software integration with EMR and CPOE system	No	No	Yes
Barcode verification of products	No	Manual	Automated
Automated registration of product deviations	No	No	Yes
Double-checking procedure			
Elements	Prescription, products	Prescription, protocol, products, weight labels	Protocol, products, weight labels
Staff type	Nurse	Pharmacy technician Pharmacist	Pharmacy technician Pharmacist
Automated registration workflow in HiX	No	No	Yes

*HiX Version 6.1 (ChipSoft B.V., Amsterdam, the Netherlands) and Practocol Version 2.0.8.2 (Practocol B.V., Rotterdam, the Netherlands) for medication in chemotherapy protocols (e.g., granisetron).

^†^TgMed Version 5.0 (Richmond B.V., Hengelo, the Netherlands).

LAF, laminar air flow.

### Intervention

The intervention consisted of centralizing the preparation of multistep intravenous admixtures in the hospital pharmacy’s CIVAS unit. Admixtures were defined as multistep if at least 1 of the following criteria was met: (1) preparation using an injection powder, (2) preparation with 3 or more medication vials, (3) syringe preparation after diluting injection liquids or infusion liquids, or (4) preparation of individual dosages requiring complex calculations. Admixtures were prepared by pharmacy staff members, mostly pharmacy technicians. After the admixtures were prepared, the double-check was performed by a pharmacy technician. Subsequently, a pharmacist reviewed the preparation protocol to approve or reject the admixture preparation. A pharmacist was always available in the CIVAS unit for questions regarding admixture preparation.

**FIGURE 1:**
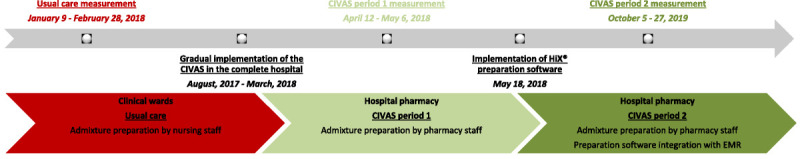
Characteristics of intervention implementation and measurements periods.

The CIVAS was gradually implemented by the hospital pharmacy in close collaboration with nursing management staff from August 2017 to March 2018 in all adult clinical wards of our institution. Two CIVAS measurement periods were examined, that is, CIVAS period 1 (CIVAS1; 1 month after implementation) and period 2 (CIVAS2; 18 months after implementation). On May 18, 2018, the hospital pharmacy replaced TgMed preparation software (Richmond B.V., Hengelo, the Netherlands) with HiX preparation software (ChipSoft B.V., Amsterdam, the Netherlands), which is integrated with the electronic medical record (EMR) system and computerized physician order entry (CPOE) system of HiX (Table 1). A timeline and brief overview of the interventions are shown in Figure 1. Nursing staff satisfaction measurements were performed 5 and 18 months after CIVAS implementation to allow nurses to adapt to the new procedures.

### Inclusion and Exclusion Criteria

Multistep intravenous admixtures intended for adult inpatients were included. Exclusion criteria were intravenous admixtures not having been completed during the observation or already having been prepared centrally before CIVAS implementation. Admixtures that could not be linked to a specific patient, medication name, or an admixture-specific preparation protocol of the hospital pharmacy were excluded for the assessment of IAPE-related outcomes.

### Definition and Classification of IAPE

An IAPE was defined as any error in the preparation of an intravenous admixture, that is, a deviation from the medication order, a deviation from the local electronic admixture preparation instructions for clinical wards, a deviation from the hospital pharmacy’s admixture-specific preparation protocol, or a deviation from the medication information sheet provided by the manufacturer if a local protocol was not available.^5,21^ Intravenous admixture preparation errors were classified by type as follows^21^: (1) wrong drug, (2) wrong dose, (3) wrong solvent or diluent, (4) wrong volume of solvent or diluent, (5) wrong infusion fluid, (6) wrong volume of infusion fluid, (7) wrong preparation technique (e.g., incomplete mixing), and (8) other. For wrong dose, wrong volume of solvent or diluent, and wrong volume of infusion fluid, deviations of more than 10% were considered erroneous. This threshold was chosen because a maximum of 10% deviation from the declared dose within the shelf-life is widely accepted for pharmaceutical products, for example, by manufacturing guidelines,^22^ and because a deviation of up to 10% is expected to be without clinical consequences. The error type incomplete mixing (i.e., container inverted less than 3 times or not shaken) occurred in the vast majority of admixture preparations (n = 1334; 82.0%). This error type was excluded because it was considered irrelevant because spontaneous mixing occurs during normal handling, especially with antibiotic admixtures (82% of the included admixtures).^23,24^

The potential severity of IAPEs was classified according to the National Coordinating Council for Medication Error Reporting and Prevention (NCC MERP) severity index,^25^ which ranges from category A (circumstances or events that have the capacity to cause error) to I (an error occurred that may have contributed to or resulted in the patient’s death).

### Nursing Staff Satisfaction Questionnaire

We adapted and translated a validated questionnaire on satisfaction with automated dispensing cabinets and replaced automated dispensing cabinets by CIVAS^26^ because validated questionnaires on CIVAS are lacking. To maximize the face validity of the resulting questionnaire, 2 nurses reviewed it on clarity, after which pilot measurements were made. The questionnaire consisted of general nursing staff characteristics (i.e., ward, sex, age, degree type, educational level, and experience), a question concerning overall satisfaction, 7 statements, and an open-ended question. The 7 statements covered nurses’ perception in the following domains: (1) efficiency of CIVAS, (2) safety of CIVAS, (3) time between request and delivery of CIVAS medication, (4) CIVAS product range, (5) number of telephone calls regarding missing CIVAS medication, (6) support by pharmacy staff, and (7) training on CIVAS. Responses were given on a 6-point Likert scale from 1 (strongly disagree) to 6 (strongly agree). Nurses indicated overall satisfaction on a visual analog scale from 0 (dissatisfied) to 100 (satisfied). The open-ended question invited remarks or suggestions on CIVAS. The questionnaire is shown in online supplementary appendix A (http://links.lww.com/JPS/A488).

### Outcomes

The primary outcome was the proportion of intravenous admixtures with 1 or more IAPEs (CIVAS2 versus clinical wards). Secondary outcomes were the proportion of intravenous admixtures with 1 or more IAPEs (CIVAS1 versus clinical wards); the type and potential severity of IAPEs (CIVAS1 and CIVAS2 versus clinical wards); rates of noncompliance to hygiene, double-checking, and labeling procedures (CIVAS1 and CIVAS2 versus clinical wards); and nursing staff satisfaction with CIVAS (18 months versus 5 months after intervention). Noncompliance rates were quantified as the proportion of intravenous admixtures with 1 or more deviations from the respective procedures.

### Data Collection

Data on the preparation of intravenous admixtures were collected by the disguised observation method.^27–29^ To minimize a Hawthorne effect, nursing staff and pharmacy staff were informed that the purpose of the observations was to study the medication process. Trained observers accompanied nursing staff or pharmacy staff members to observe and record every admixture preparation on observation forms designed for this study. Nursing staff were asked for verbal consent before initiation of an observation. Observers were instructed to intervene only if a serious error took place (e.g., wrong patient or medication).^28^ Observation rounds of several hours (generally 4–5 hours) were planned beforehand in a period of 1 week for the neurosurgery ward and approximately 3 weeks for the hematology and internal oncology wards, CIVAS1, and CIVAS2. The observation period of the neurosurgery ward was shorter than that of the other units because of the short period between the start of our study and the implementation of the intervention on that ward. A pharmacist (J.G.J.) and hospital pharmacist/clinical pharmacologist (N.G.M.H.) independently reviewed the data on the observation forms to determine if an IAPE had occurred. If so, they determined the type and potential severity of the IAPE. Assessments were recorded on review forms; any disagreement between assessments was resolved by consensus. After completion of observation rounds in a particular setting, observed staff were asked to record their sex, age, degree type, educational level, and experience. Data on the characteristics of patients (i.e., sex, birth date, and number of prescribed medications) for whom admixture preparations were observed were collected by 1 pharmacist (J.G.J.) from HiX and Practocol (Practocol B.V., Rotterdam, the Netherlands). The complexity of the admixture preparations, medication class by Anatomical Therapeutic Chemical classification,^30^ and day of the week were assessed by 1 pharmacist (J.G.J.). Data on the time window of admixture preparation (7:00 am to 10:00 am, 10:00 am to 2:00 pm, 2:00 pm to 6:00 pm, 6:00 pm to 11:00 pm, and 11:00 pm to 7:00 am) were collected during observation. Two study members independently calculated noncompliance rates for each observation period in the hospital pharmacy based on the observed time, number of glove renewals or disinfections, and the number of included admixtures (online Supplementary Appendix B, http://links.lww.com/JPS/A488); disagreements between assessments were resolved by consensus. For nursing staff satisfaction measurements, trained students visited the clinical wards and presented the satisfaction questionnaire on an iPad (Apple Inc., Cupertino, California) to the nursing staff present.

Nursing staff satisfaction data were exported to Excel (Microsoft Corporation, Redmond, Washington), and other collected data were entered in OpenClinica Version 2.1 (OpenClinica, LLC, Waltham, Massachusetts).

### Sample Size Calculation

Inclusion of 277 observations in each measurement period would be required to identify an assumed IAPE rate reduction from 15% in usual care to 7.5% in CIVAS2,^6^ based on a χ^2^ test using a significance level of 0.05 and a power of 80%.

### Data Analysis

The IAPE rates for both CIVAS1 and CIVAS2 were compared with those of usual care using univariable and multivariable mixed-effects logistic regression analyses. The dependent variable in these models was whether an IAPE had occurred (yes or no). We adjusted for the covariates time window of admixture preparation (7:00 am to 10:00 am, 10:00 am to 2:00 pm, 2:00 pm to 6:00 pm, and 6:00 pm to 7:00 am) and day of admixture preparation (weekdays versus weekend). The analyses accounted for repeated measurements on patient and staff member levels. To make analysis possible due to correlation in a series of admixtures, only the first admixture was included in the mixed-effects logistic regression analyses for admixtures with the following 5 identical characteristics: staff member, patient, medication name, time window, and date of admixture preparation. The results of the mixed-effects logistic regression analyses are reported as adjusted odds ratios with 95% confidence intervals. The detailed statistical analysis is described in online Supplementary Appendix C (http://links.lww.com/JPS/A488). The Mann-Whitney test was used for the overall nursing staff satisfaction scores (18 months versus 5 months after intervention). For all statistical analyses, a 2-tailed *P* < 0.05 was considered statistically significant. R Statistics Version 4.0.2 (The R Foundation, Vienna, Austria) with the package lme4 was used for the mixed-effects logistic regression analyses and the mixed-effects proportional odds logistic regression analyses. IBM SPSS Statistics Version 25 (IBM Corporation, Armonk, New York) was used for other analyses.

## RESULTS

A total of 1626 admixtures were included (clinical wards, n = 282; CIVAS1, n = 796; CIVAS2, n = 543). Seven admixtures could not be linked to a specific nursing staff member and 3 not to a specific patient. Observations of 1 observer were completely excluded because of protocol deviations during observation. Table 2 lists the characteristics of included intravenous admixtures, staff members, and patients. The 10 most frequently prepared medications, which accounted for 82.2% of all included admixtures, were cefuroxime (n = 289), meropenem (n = 285), piperacillin/tazobactam (n = 151), vancomycin (n = 150), flucloxacillin (n = 143), benzylpenicillin (n = 90), pantoprazole (n = 61), colistin (n = 50), ceftriaxone (n = 44), cyclosporine (n = 41), ceftazidime (n = 33), and prednisolone (n = 33).

**TABLE 2:** Characteristics of Included Intravenous Admixtures, Staff Members, and Patients

Characteristics	Clinical Wards Usual Care	Hospital Pharmacy CIVAS Period 1	Hospital Pharmacy CIVAS Period 2
Intravenous admixtures			
Admixtures, n	285	798	543
Clinical ward, n (%)			
Hematology	212 (74.4)	—	—
Internal oncology	52 (18.2)	—	—
Neurosurgery	21 (7.4)	—	—
Pharmaceutical form medication vial, n (%)			
Injection powder	211 (74.0)	752 (94.2)	507 (93.4)
Injection liquid	57 (20.0)	46 (5.8)	34 (6.3)
Infusion	17 (6.0)	0	2 (0.4)
Medication class (ATC classification), n (%)			
Anti-infectives for systemic use (J)	207 (72.6)	666 (83.5)	453 (83.4)
Other	78 (27.4)	132 (16.5)	90 (16.6)
Day of the week, n (%)			
Weekdays	174 (61.1)	496 (62.2)	303 (55.8)
Weekend	111 (38.9)	302 (37.8)	240 (44.2)
Time window,* n (%)			
7:00 am to 10:00 am	60 (21.1)	343 (43.0)	160 (29.5)
10:00 am to 2:00 pm	76 (26.7)	314 (39.3)	198 (36.5)
2:00 pm to 6:00 pm	86 (30.2)	141 (17.7)	185 (34.1)
6:00 pm to 7:00 am	62 (21.8)	0	0
Staff members^†^			
Staff members, n	74	18	21
Staff members, personal data available, n (%)	20 (27.0)	17 (94.4)	21 (100)
Male, n (%)	3 (4.1)	0	2 (9.5)
Age, median (IQR)	35 (27–49)	29 (25–38)	26 (22–38)
Degree type, n (%)			
Nurse	11 (55.0)	—	—
Specialized nurse	9 (45.0)	—	—
Pharmacy technician	—	17 (100)	19 (90.5)
Student (other)	—	0	2 (9.5)
Educational level, n (%)			
Secondary vocational education	10 (50.0)	16 (94.1)	18 (85.7)
Higher professional education	10 (50.0)	1 (5.9)	0
University education	0	0	2 (9.5)
Other	0	0	1 (4.8)
Experience since diploma,^‡^ n (%)			
0–1 y	0	4 (23.5)	3 (14.3)
1–5 y	5 (25.0)	6 (35.3)	8 (38.1)
>5 y	15 (75.0)	7 (41.2)	6 (28.6)
No diploma	0	0	4 (19.0)
Patients^§^			
Patients, n	67	189	211
Male, n (%)	40 (59.7)	111 (58.7)	122 (57.8)
Age, median (IQR), y	60 (51–67)	61 (47–69)	61 (48–70)
Prescribed medications per day, median (IQR)	14 (11–18)	13 (10–17)	13 (9–17)

*Missing time window: 1 intravenous admixture in clinical wards.

^†^Missing nursing staff identifier: 7 intravenous admixtures.

^‡^Experience since graduation from the nursing or pharmacy technician training program.

^§^Missing patient identifier: 3 intravenous admixtures.

ATC, Anatomical Therapeutic Chemical.

### Intravenous Admixture Preparation Errors

Of the 1626 included admixtures, 5 (0.3%) were excluded for IAPE analyses because of missing data (n = 3, patient identifier; n = 2, admixture-specific preparation protocol). Observers did not intervene in any admixture preparation because of a serious error.

#### Prevalence of IAPEs

Intravenous admixture preparation error prevalences and effects of CIVAS on IAPEs are quantified in Table 3. One or more IAPEs were identified in 14 of 543 admixtures (2.6%) in CIVAS2, in 63 of 796 admixtures (7.9%) in CIVAS1, and in 148 of 282 admixtures (52.5%) in the clinical wards (CIVAS2: adjusted odds ratio, 0.02; 95% confidence interval, 0.004–0.05; CIVAS1: adjusted odds ratio, 0.06; 95% confidence interval, 0.02–0.18).

**TABLE 3:** Effects of a Pharmacy-based CIVAS on IAPEs

	IAPE Prevalence		Mixed-Effects Logistic Regression Analysis*^†^	
	All Admixtures, n/N (%)	After Exclusion of Matched Admixtures*, n/N (%)	Univariable Analysis (n = 995), Odds Ratio (95% Confidence Interval)	Multivariable Analysis^‡^ (n = 994), Adjusted Odds Ratio (95% Confidence Interval)
Measurement period				
Clinical wards: usual care	148/282 (52.5)	134/264 (50.8)	Reference	Reference
Hospital pharmacy: CIVAS period 1	63/796 (7.9)	28/319 (8.8)	**0.06 (0.02–0.16)**	**0.06 (0.02–0.18)**
Hospital pharmacy: CIVAS period 2	14/543 (2.6)	11/419 (2.6)	**0.02 (0.005–0.05)**	**0.02 (0.004–0.05)**

*Only the first admixture was included for matched admixtures, that is, admixtures with the following identical characteristics: staff member, patient, medication name, time window, and date of admixture preparation.

^†^Mixed-effects logistic regression analysis was used to account for within-subject correlations because of repeated measurements by staff members and patients.

^‡^Odds ratios have been adjusted for time window and day of admixture preparation.

Odds ratios in bold have a *P* < 0.05.

#### Type and Severity of IAPEs

Overall, the most common IAPE types were wrong solvent or diluent (n = 107; 6.6%) and wrong volume of infusion fluid (n = 69; 4.2%). The type and severity of IAPEs stratified by clinical ward, CIVAS1, and CIVAS2 are shown in Table 4.

**TABLE 4:** Type and Severity of IAPEs in Clinical Wards and the Hospital Pharmacy

	Clinical Wards Usual Care	Hospital Pharmacy CIVAS Period 1	Hospital Pharmacy CIVAS Period 2
Intravenous admixtures, n	282	796	543
Admixtures with ≥1 IAPEs, n (%)	148 (52.2)	63 (7.9)	14 (2.6)
IAPEs, n	173	63	15
Type of IAPEs, n (%)			
Wrong drug	2 (0.7)	0	0
Wrong dose	9 (3.2)	16 (2.0)	1 (0.2)
Wrong solvent or diluent	87 (30.9)	12 (1.5)	8 (1.5)
Wrong volume of solvent or diluent	18 (6.4)	5 (0.6)	0
Wrong infusion fluid	8 (2.8)	1 (0.1)	0
Wrong volume of infusion fluid	43 (15.2)	24 (3.0)	2 (0.4)
Wrong preparation technique	5 (1.8)	3 (0.4)	4 (0.7)
Incomplete powder dissolution	5	3	4
Other	1 (0.4)	2 (0.3)	0
Severity of IAPEs,* n (%)			
Potential error			
A	0	2 (0.3)	0
No harm			
C/D	151 (53.5)	47 (5.9)	15 (2.8)
Harm			
E	20 (7.1)	14 (1.8)	0
F	1 (0.4)	0	0
H	1 (0.4)	0	0

*NCC MERP classification: no error (category A); error, no harm (categories B to D); error, harm (categories E to H); and error, death (category I). A: circumstances or events that have the capacity to cause error; C: an error occurred that reached the patient but did not cause patient harm; D: an error occurred that reached the patient and required monitoring to confirm that it resulted in no harm to the patient and/or required intervention to preclude harm; E: an error occurred that may have contributed to or resulted in temporary harm to the patient and required intervention; F: an error occurred that may have contributed to or resulted in temporary harm to the patient and required initial or prolonged hospitalization; H: an error occurred that required intervention necessary to sustain life.

The prevalence of potentially harmful IAPEs, that is, errors classified in NCC MERP category E or higher, in CIVAS1 (n = 14; 1.8%) and CIVAS2 (n = 0; 0.0%) was lower than that in clinical wards (n = 22; 7.8%).

### Noncompliance to Hygiene, Double-checking, and Labeling Procedures

Compared with clinical wards, CIVAS1 and CIVAS2 had lower rates of noncompliance to hygiene procedures regarding the disinfection of workbenches, disinfection of vials, and double-checking of the preparation and had comparable rates of noncompliance to glove use and admixture labeling (Table 5).

**TABLE 5:** Rates of Noncompliance to Hygiene, Double-checking, and Labeling Procedures

Preparation Procedures	Clinical Wards Usual Care, Admixtures With a Deviation, n/N (%)	Hospital Pharmacy CIVAS Period 1, Admixtures With a Deviation, n/N (%)	Hospital Pharmacy CIVAS Period 2, Admixtures With a Deviation, n/N (%)
Hygiene procedures			
Workbench disinfection	149/275 (54.2)	0/796 (0)	0/537 (0)
Hand disinfection	187/250 (74.8)	Not applicable	Not applicable
Glove use	2/284 (0.7)	0/798 (0)	0/541 (0)
Glove renewal	Not applicable	69/719 (9.6)	127/543 (23.4)
Glove disinfection*	Not applicable	84/369 (22.8)	207/318 (65.1)
Adequate sequence of hand-hygiene measures	84/280 (30.0)	Not applicable	Not applicable
Vial disinfection	117/276 (42.4)	97/783 (12.4)	66/523 (12.6)
Multiple-vial penetration	11/285 (3.9)	Not applicable	Not applicable
Double-checking procedures	146/285 (51.2)	25/796 (3.1)	6/525 (1.1)
Labeling procedures	1/283 (0.4)	0/788 (0)	0/542 (0)

*Disinfection of gloves was not applicable in 350 admixtures in CIVAS period 1 and 225 admixtures in CIVAS period 2.

### Nursing Staff Satisfaction

A total of 286 nurses completed the satisfaction questionnaire. Characteristics of questionnaire participants are shown in online Supplementary Appendix D (http://links.lww.com/JPS/A488). Overall satisfaction with the CIVAS increased from a median of 70 (interquartile range [IQR], 62–78; n = 166) 5 months after intervention to 77 (IQR, 71–83; n = 115) 18 months after intervention (*P* < 0.001). After 18 months, the scores of the 7 statements on a 6-point Likert scale were as follows: (1) efficiency of CIVAS, 5 (IQR, 5–5; n = 118); (2) safety of CIVAS, 5 (IQR, 4–5; n = 118); (3) time between request and delivery of CIVAS medication, 4 (IQR, 4–5; n = 118); (4) CIVAS product range, 5 (IQR, 4–5; n = 117); (5) number of telephone calls regarding missing CIVAS medication, 4 (IQR, 3–5; n = 118); (6) support by pharmacy staff, 5 (IQR, 4–5; n = 118); and (7) training on CIVAS, 5 (IQR, 5–6; n = 118).

The responses to the open-ended question consisted of varying remarks and suggestions but were mainly related to expanding the admixture product range and optimizing delivery times.

## DISCUSSION

The implementation of the pharmacy-based CIVAS was associated with a much lower probability of IAPEs (odds ratio, 0.02). The implementation of the CIVAS also resulted in lower rates of potentially harmful IAPEs and of noncompliance to several hygiene procedures (workbench disinfection and vial disinfection) and double-checking procedures. In addition, nursing staff satisfaction with the CIVAS was high.

The CIVAS had lower rates of all IAPE types, including wrong-drug and wrong-dose errors, and reduced the rates of potentially harmful IAPEs (NCC MERP category E or higher) from 7.8% to 1.8% in CIVAS1 and 0.0% in CIVAS2.

In contrast to CIVAS1, during CIVAS2, a preparation software with an automated barcode verification of products and an automated workflow with regard to documentation was used. This new software was also integrated with the EMR and CPOE of the same manufacturer. Compared with CIVAS1, CIVAS2 showed a further reduction of at least 0.5% of the following error types: wrong dose (1.8% absolute reduction), wrong volume of solvent or diluent (0.6% absolute reduction), and wrong volume of infusion fluid (2.6% absolute reduction). This may be attributed to the new features. For example, barcode verification may prevent errors related to wrong dose, wrong drug/solvent/infusion fluid, or wrong volume of infusion fluid. In addition to the IAPE measurements, we examined the extent of noncompliance to hygiene procedures. As expected, compared with the clinical wards, the CIVAS unit had lower rates of noncompliance to several hygiene procedures, that is, workbench disinfection and vial disinfection. Nonetheless, substantial rates of noncompliance to several hygiene procedures were identified in both the clinical wards and the CIVAS unit and therefore warrant further attention.

The high IAPE rates in clinical wards and procedural noncompliance rates merit exploration of potential causes. Previous studies have indicated many potential causes of medication preparation and administration errors, such as lack of knowledge, high workload, distractions, and inadequately written communication, but studies on IAPEs in particular are scarce.^31–35^ With regard to risk factors of IAPEs in clinical wards, a study at our institution showed that especially multistep preparations (versus single-step preparations), interruptions during preparation, weekend preparations (versus weekday preparations), time window 2:00 pm to 6:00 pm (versus 7:00 am to 10:00 am), and preparation in adult wards (versus pediatric wards) were associated with an increased probability of IAPEs.^36^ To examine the causes of the IAPEs and noncompliance to hygiene procedures, robust qualitative studies are needed.

Unfortunately, the results of this intervention study cannot be directly compared with the results of previous centralization studies,^13–16^ which were performed in dissimilar settings (i.e., the intensive care unit^13,14,16^), used nonobservational techniques (i.e., microbial determinations^15,16^ or laboratory measurements^13,14^), or investigated limited types of IAPEs^13–16^ (i.e., concentration errors and calculation errors).

This study has several strengths. First, we collected data on the preparation of intravenous admixtures intended for administration to inpatients performed by a large number of staff members, thereby supporting the generalizability of the results of this study. Furthermore, we did not limit the types of IAPEs for inclusion and used a robust method to estimate the presence, type, and potential severity of IAPEs. This study also has limitations. First, observer bias may have occurred, even though the observation method used is the gold standard to detect medication errors.^27,29^ We have taken many measures to limit observer bias, such as using the disguised observation method and extensive training of observers. Any errors due to observer bias will have occurred randomly across the 3 measurement periods. Also, the high error rate identified in this study suggests a negligible Hawthorne effect. Second, our findings are based on data from 1 university hospital, potentially limiting the generalizability. Third, we did not use a validated questionnaire to estimate nursing staff satisfaction with CIVAS because such a questionnaire was lacking. Lastly, before-and-after studies are inherently susceptible to bias because of the lack of randomization.

Taken together, our findings support the implementation of a CIVAS to improve patient safety. Our findings also suggest that the compliance to hygiene and double-checking deserves further attention. Future research should focus on analyzing the cost-effectiveness and sustainability of CIVAS systems because extensive resources are necessary to implement CIVAS in a hospital. Necessary investments included those related to additional pharmacy space, clean room facilities and materials, technology (e.g., additional software, scanners, computers), maintenance and support, and pharmacy personnel. However, pharmacy technicians are generally cheaper than nurses, and CIVAS may lead to improved compliance with formularies, improved stock management (traceability and accountability), and decreased wastage (e.g., by extended expiry dates).^37,38^ Also, future qualitative studies should explore the causes of IAPEs and noncompliance to hygiene procedures to develop potentially more efficient solutions for error prevention.

## CONCLUSIONS

The implementation of CIVAS for multistep preparations was associated with a substantially decreased probability of IAPEs, including potentially harmful ones. In addition, CIVAS performed significantly better than clinical wards with regard to compliance to hygiene and double-checking procedures. Nurses were satisfied with CIVAS. Hence, CIVAS implementation is an important strategy to improve medication safety in hospitals.

## Supplementary Material
