# Supplementary material for: Effect of a Pharmacy-based Centralized Intravenous Admixture Service on the Prevalence of Medication Errors: A Before-and-After Study
Source: J Patient Saf. 2022 Jun 30;18(8):e1181–8. doi: 10.1097/PTS.0000000000001047 (PMC9698191; doi:10.1097/PTS.0000000000001047)
Supplement: Supplementary file 1 [file jps-18-e1181-s001.docx]

**SUPPLEMENTARY MATERIALS**

### Appendix A Nursing staff satisfaction questionnaire (English translation)

*This questionnaire concerns the newly implemented centralized intravenous admixture service from the hospital pharmacy. The hospital pharmacy now delivers multistep intravenous admixtures to your clinical ward. Before that, nursing staff themselves prepared intravenous admixtures. We would like to know how satisfied you are, and what your experiences are, with this new service. We would be pleased if you would be so kind to fill in this short questionnaire.*

| *Ward name* |  |
| --- | --- |
| *Initials and surname* |  |
| *Age in years* |  |
| *Gender* | Male  Female |
| *Educational level* | Secondary vocational education  Higher professional education  University education  Other:……… |
| *Job position* | Student nurse  Nurse  Specialised nurse  Other:…. |
| *Work experience since nursing diploma registration* | Less than year  1 to 5 years  More than 5 years |
| *Work experience in healthcare settings* | Less than 1 year  1 to 5 years  More than 5 years |

*Scale 1-6 (1= strongly disagree, 6= strongly agree)*

| 1 | The centralized intravenous admixture service makes administering medication more efficient for me | 1, 2, 3, 4, 5, 6 |
| --- | --- | --- |
| 2 | The centralized intravenous admixture service makes it easier for me to work more safely | 1, 2, 3, 4, 5, 6 |
| 3 | The time elapsed between requesting and receiving intravenous admixtures is acceptable | 1, 2, 3, 4, 5, 6 |
| 4 | The number of types of intravenous admixtures available is acceptable | 1, 2, 3, 4, 5, 6 |
| 5 | The number of telephone calls to the pharmacy to request missing intravenous admixtures is acceptable | 1, 2, 3, 4, 5, 6 |
| 6 | Pharmacy staff is helpful when answering my questions and resolving problems | 1, 2, 3, 4, 5, 6,  Not applicable |
| 7 | The training session was sufficiently informative | 1, 2, 3, 4, 5 , 6,  Not applicable |
|  | | |
| **8** | **How satisfied are you with the new centralized intravenous admixture service?** | |
|  | Dissatisfied 0 100 Satisfied | |
| **9** | **Remarks and suggestions**  ***Safety***  ***Ease of use***  ***Product range***  ***Other*** | |

### Appendix B Calculation of rates of non-compliance to glove renewal and glove disinfection in the hospital pharmacy

Non-compliance rates were calculated for each observation period with the following formulas:

$\left( 1-\frac{performed number of glove renewals}{expected number of glove renewals} \right)x 100\%$

$\left( 1-\frac{performed number of glove disinfections}{\left( \mathrm{adjusted} \right)expected number of glove disinf\mathrm{ections}} \right)x 100\%$

The expected number of glove renewals and glove disinfections depended on the length of the observation period. For example, for an observation period of 65 minutes, the expected number of glove renewals was two, i.e. after 30 and 60 minutes, and of glove disinfections was two, i.e. after 15 and 45 minutes. The number of glove renewals that were additional to the expected number of glove renewals were subtracted from the expected number of glove disinfections, i.e. the adjusted expected number of glove disinfections; whereas, the number of glove renewals that were subtractive were added to the expected number of glove disinfections.

If glove renewal took place, glove disinfection was marked as not applicable to admixtures in the next 15 minutes; the number of admixtures for which glove disinfection was applicable was calculated with the following formula:

$\frac{expected number of glove disinfections}{performed number of glove renewals+expected number of glove disinfections} x total number of admixtures$

Allocation to the total number of included admixtures within the related observation was based on calculated non-compliance rates.

###

### Appendix C Detailed data analysis

The IAPE rates for both CIVAS1 and CIVAS2 were compared to usual care using univariable and multivariable mixed-effects logistic regression analysis (i.e. generalized linear mixed models). The dependent variable in these models was a dichotomous variable indicating whether an IAPE occurred (categorized; yes, no); the independent variables were setting (categorized; clinical wards, CIVAS1, CIVAS2) and the covariates time window of admixture preparation (categorized; 7 a.m.-10 a.m., 10 a.m.-2 p.m., 2 p.m.-6 p.m., 6 p.m.-7 a.m.) and day of admixture preparation (categorized; weekdays, weekend). Because of data dependence and the low number of non-events, a selection of covariates was made based on theoretical and reported associations^1-3^. The significance of the covariates was confirmed in a subsequent study at our institution that showed time window and day of the week were significantly associated with the occurrence of IAPEs.^4^

To account for repeated measurements and the within-subject correlations, we included two random effects, namely a random intercept by staff member and a random intercept by patient, leading to a model with crossed random effects. We also tested whether the variance of the random intercept of the staff members differed between nurses (clinical wards) and pharmacy technicians (CIVAS1 and CIVAS2), and included this difference in variance parameters in the model when statistically significant. For the multivariable analyses, complete case analyses were performed. The results of the mixed-effects logistic regression analyses are reported as adjusted odds ratios with 95% confidence intervals. In preliminary analyses, no convergence of parameter estimates was obtained in the mixed-effects logistic regression models due to large within-patient dependence. The cause of this problem was identified as the occurrence of many similar or identical admixtures for the same patient on the same day. Typically, when such a series of admixtures is performed, an IAPE is made for either all or none of the admixtures, leading to correlation in the repeated measurements. To remedy this problem, only the first admixture was included in the mixed-effects logistic regression analyses for admixtures with the following five identical characteristics: staff member, patient, medication name, time window and date of admixture preparation.

The Mann-Whitney *U* test was used for the overall nursing staff satisfaction scores (18 months versus 5 months post-intervention).

For all statistical analyses, a two-tailed *p* value < .05 was considered statistically significant. R Statistics® version 4.0.2. (The R Foundation; Vienna, Austria) with the package lme4 was used for the mixed-effects logistic regression analyses and the mixed-effects proportional odds logistic regression analyses. IBM SPSS Statistics® version 25 (IBM Corporation; Armonk, New York, United States) was used for other analyses.

1. Deng Y, Lin AC, Hingl J, et al. Risk factors for i.v. compounding errors when using an automated workflow management system. Am J Health Syst Pharm 2016; 73: 887-93.

2. Ong WM, Subasyini S. Medication errors in intravenous drug preparation and administration. Med J Malaysia 2013; 68: 52-7.

3. Nguyen HT, Nguyen TD, van den Heuvel ER, et al. Medication Errors in Vietnamese Hospitals: Prevalence, Potential Outcome and Associated Factors. PLoS One 2015; 10: e0138284.

4. Jessurun JG, Hunfeld NGM, van Rosmalen J, et al. Prevalence and determinants of intravenous admixture preparation errors: A prospective observational study in a university hospital. Int J Clin Pharm 2022; 44: 44-52.

### Appendix D Characteristics of questionnaire participants on nursing staff satisfaction with the centralized intravenous admixture service (CIVAS)

| ***Characteristics*** | ***5 months***  ***after CIVAS implementation*** | ***18 months***  ***after CIVAS implementation*** |
| --- | --- | --- |
| Participants, n | *168* | *118* |
| Clinical ward, n (%)  Hematology  Internal oncology  Neurosurgery  Hepatopancreatobiliary surgery  Pulmonary medicine  Neurology  Other | 37 (22.0)  25 (14.9)  20 (11.9)  34 (20.2)  19 (11.3)  20 (11.9)  13 (7.7) | 36 (30.5)  19 (16.1)  14 (11.9)  18 (15.3)  17 (14.4)  9 (7.6)  5 (4.2) |
| Male, n (%) | 11 (6.5) | 13 (11.0) |
| Age, median (IQR) | 27 (24-36) | 26 (23-43) |
| Job position, n (%)  Nurse  Specialized nurse  Student nurse  Other | 122 (72.6)  29 (17.3)  11 (6.5)  6 (3.6) | 82 (69.5)  25 (21.2)  10 (8.5)  1 (0.8) |
| Educational level, n (%)  Secondary vocational education  Higher professional education  University education  Other  Experience since diploma, n (%)  0 to 1 year  1 to 5 years  More than 5 years | 67 (39.9)  94 (56.0)  1 (0.6)  5 (3.0)  41 (24.4)  48 (28.6)  79 (47.0) | 53 (44.9)  59 (50.0)  1 (0.8)  4 (3.4)  25 (21.2)  42 (35.6)  48 (40.7) |

IQR, interquartile range
